# Enhancing *Reuse* of Data and Biological Material in Medical Research: From FAIR to FAIR-Health

**DOI:** 10.1089/bio.2017.0110

**Published:** 2018-04-01

**Authors:** Petr Holub, Florian Kohlmayer, Fabian Prasser, Michaela Th. Mayrhofer, Irene Schlünder, Gillian M. Martin, Sara Casati, Lefteris Koumakis, Andrea Wutte, Łukasz Kozera, Dominik Strapagiel, Gabriele Anton, Gianluigi Zanetti, Osman Ugur Sezerman, Maimuna Mendy, Dalibor Valík, Marialuisa Lavitrano, Georges Dagher, Kurt Zatloukal, GertJan B. van Ommen, Jan-Eric Litton

**Affiliations:** ^1^ BBMRI-ERIC, Graz, Austria.; ^2^ Technical University of Munich, Munich, Germany.; ^3^ TMF e.V., Berlin, Germany.; ^4^ BBMRI.mt and University of Malta, Msida, Malta.; ^5^ BBMRI.it and Universita degli Studi di Milano-Bicocca, Milano, Italy.; ^6^ BBMRI.gr and Foundation for Research and Technology-Hellas, Heraklion, Greece.; ^7^ BBMRI.pl and Wroclaw Research Centre EIT+, Wroclaw, Poland.; ^8^ BBMRI.pl and University of Łódź, Łódź, Poland.; ^9^ Helmholtz Zentrum München, Munich, Germany.; ^10^ BBMRI.it and CRS4, Pula, Italy.; ^11^ BBMRI.tr and Acibadem University, Istanbul, Turkey.; ^12^ BBMRI.IARC and International Agency for Research on Cancer, Lyon, France.; ^13^ BBMRI.cz and Masaryk Memorial Cancer Institute, Brno, Czech Republic.; ^14^ INSERM, Paris, France.; ^15^ BBMRI.at and Medical University Graz, Graz, Austria.; ^16^ BBMRI.nl and Leiden University Medical Center, Leiden, Netherlands.

**Keywords:** FAIR (Findable, Accessible, Interoperable, and Reusable) principles, provenance information management, privacy protection, open science, quality, incentives

## Abstract

The known challenge of underutilization of data and biological material from biorepositories as potential resources for medical research has been the focus of discussion for over a decade. Recently developed guidelines for improved data availability and reusability—entitled FAIR Principles (Findability, Accessibility, Interoperability, and Reusability)—are likely to address only parts of the problem. In this article, we argue that biological material and data should be viewed as a unified resource. This approach would facilitate access to complete provenance information, which is a prerequisite for reproducibility and meaningful integration of the data. A unified view also allows for optimization of long-term storage strategies, as demonstrated in the case of biobanks. We propose an extension of the FAIR Principles to include the following additional components: (1) quality aspects related to research reproducibility and meaningful reuse of the data, (2) incentives to stimulate effective enrichment of data sets and biological material collections and its reuse on all levels, and (3) privacy-respecting approaches for working with the human material and data. These FAIR-Health principles should then be applied to both the biological material and data. We also propose the development of common guidelines for cloud architectures, due to the unprecedented growth of volume and breadth of medical data generation, as well as the associated need to process the data efficiently.

## Introduction

**FIG. 1.**
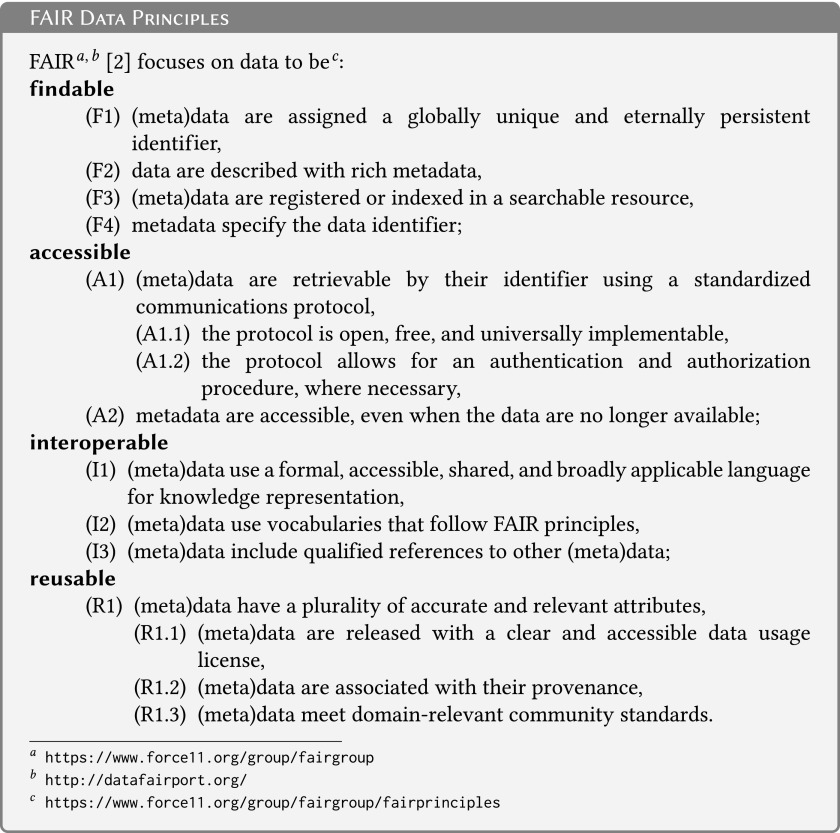
FAIR data principles.

Inefficient sharing of data^1^ generated from public funding and increasing dependence of research domains on data have led to the development of specific guidelines such as the FAIR data principles: Findable, Accessible, Interoperable, and Reusable.^2^ While the FAIR principles (Fig. 1) are a good starting point, applicable to various domains of science, they are not specific enough to deal with the major challenges of medical research, namely *reproducibility and privacy protection*. We propose a unified view of biological material and data together with specific extensions to the FAIR data principles, to boost their use and reuse in medical research.

These FAIR-Health principles include the following three main components: (1) *quality and traceability*; (2) *incentive schemes*; and (3) *privacy regulation compliance*. FAIR-Health principles are also applicable to other fields dealing with health-related and sensitive personal data, such as social sciences.

## Biobanks: A Unified View of Biological Material, Expertise, and Data

**FIG. 2.**
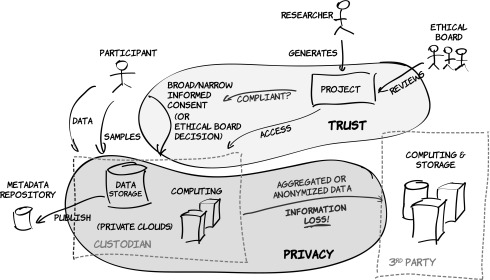
The flow of data and samples from the research participants to biobanks, and then to researchers. Note the particular aspect of matching informed consent provided by the research participant to the research project, which can also be substituted by an ethical board decision if consent is not available.

The European Biobanking and BioMolecular resources Research Infrastructure, BBMRI-ERIC,* anticipates substantial potential in the ability of biobanks to completely integrate the chain, from research participants and their data/samples all the way to research results (Fig. 2).

Biobanks consist of the following key components: (1) *repositories of biological material* retrieved as samples from the research participants; (2) *metadata describing repositories and stored samples*; (3) *data accompanying the samples* (medical records, including imaging data and lifestyle data); (4) *data generated from the samples* (e.g., omics data and imaging data); (5) *expertise* in various fields related to long-term preservation and analyses of biological material and data, ethical/legal expertise; and (6) *additional services* related to biobanks as infrastructure (e.g., sample hosting, processing, and curation).^3–5^ Many biobanks are intended as facilities for long-term use, and thus research can additionally benefit from the extensive possibilities of longitudinal sample and data acquisition, such as samples/data from the same donor at different time points for studies concerning disease or treatment markers.

With current analytic and data collection techniques capable of generating almost unlimited amounts of data on each research participant donating samples and data, the question naturally arises as to whether it is meaningful, sustainable, and even technically feasible to collect, validate, store, and curate all the data on a long-term basis, and whether the volume of data is what will make medical and biomedical research more productive in the future.

As part of the strategy to address these issues, *BBMRI-ERIC proposes a unified view of biological material and the data generated from the material*, since biological material can be considered biological data storage/a biological data source. The biological material and data custodians can thus apply different strategies when combining storage to achieve acceptable costs: large raw data may be understood as transient data that can be regenerated if the original material is still available and its integrity is preserved in the long-term storage; hence, only relatively small resulting data may be kept together with the original material. We additionally argue that the high-level principles of FAIR-Health should also be applied to the biological material. The only specific aspects of biological material are that it varies in its properties and is *depletable* (except for amplifiable derivatives, such as cell cultures or DNA), and therefore of limited accessibility (namely subject to access prioritization).

Translation of research results to medically meaningful knowledge and products suffers from substantial reproducibility issues.^6–13^ Recently, this has stimulated the proposal of countermeasures that are technically infeasible, such as an idea to store all the intermediate data used for publications^14, Rule 5^. Although recognizing that primary biological material and data have to be stored for the sake of “reconstructing” results, which need to comply with principles of Good Research Practice,^†^ it is, however, not practical and sometimes not even feasible to store all the data, due to the sheer volume of all the intermediate data. It can even be argued that storing intermediate data is of limited use when exploring reproducibility of the results, for example because of undocumented or proprietary formats.

**FIG. 3.**
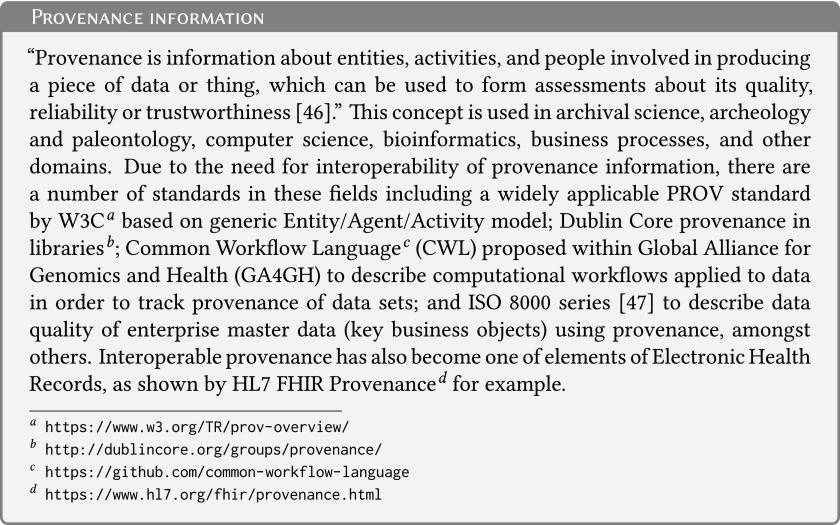
Provenance information.

Furthermore, *reproducibility*^15^ needs a clear link from the research participant (or other sources of nonhuman biological material), through the physical material and all the preanalytical steps, including sample stabilization and biobanking, to the data generated and published. Such links can be implemented using provenance information management systems^16^ (see Fig. 3 for background information on provenance). Incorporation of the preanalytical steps as a part of data provenance is increasingly recognized^17^ because the preanalytical phase has major implications on the preservation of biomolecules, and thus on *meaningfulness* of the data generated by the analysis of biological samples. For example, RNA analysis of a biological material can be subject to the checks of integrity of RNA molecules and thus the overall conclusion may be that the results are meaningful. However, the biological material, which is still “alive,” may have responded to the artificial environmental conditions after its removal from its original environment, such as after a surgical resection of a tumor^‡^^18, Annex A^.

**FIG. 4.**
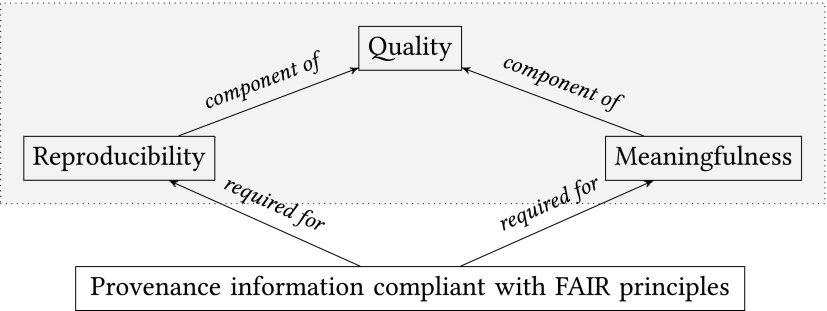
Relationship between provenance information and components of quality for biological material and data.

As a consequence, the material analyzed no longer represents the original material and its biological activity in the human body. While the analysis of such material is then performed in a technically correct manner, the results might not be meaningful. Accordingly, a prerequisite to obtain meaningful data requires an assessment of fitness of the biological material, based on the provenance information, for the purpose of the specific research and analytical method. The reproducibility and the meaningfulness jointly determine the *quality of the biological material and data* (Fig. 4). The advantage of infrastructures providing a unified view of physical material and data, such as biobanks, is that they can integrate provenance information naturally.

As part of quality improvement efforts, it is also necessary to document syntax, semantics, and history of data as completely as possible. This is particularly important for types of data that have been collected primarily for the purpose of healthcare, but can still be reused for research as a “secondary use,” namely lawful use that requires fulfilling specific regulatory requirements, such as specifying anticipated secondary uses in the informed consent, or obtaining approval from an ethics committee for the reuse of data under specific conditions. While the data generation adheres to medical and laboratory best practices at any given moment, technology advances, methods evolve, and instruments are upgraded to ensure the best standard of healthcare over time.

These changes may result in vast problems when integrating the data into consistent data sets, particularly as semantics of data changes over time and across various sources, such as biorepositories, cohorts, or laboratories. These problems stimulate the need for data models and formats that can unambiguously capture syntax and semantics of the data, such as numeric data with defined notation, units, and semantics of the data, as well as methods used to generate such data.^§^

### Provenance information management

**FIG. 5.**
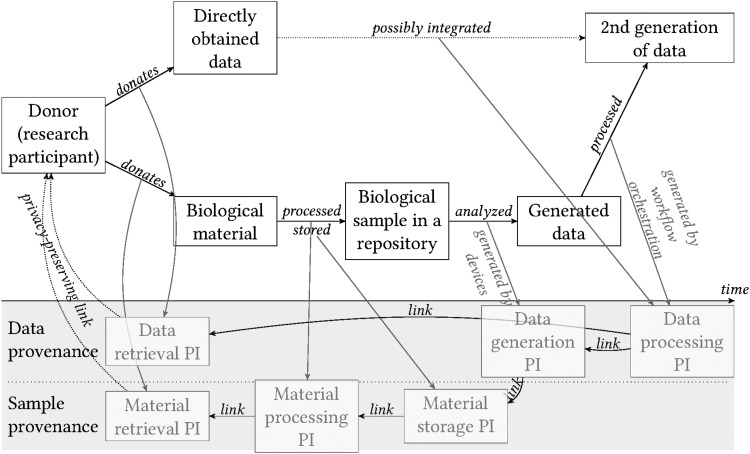
The ideal complete coverage of provenance information for biological material and associated data. Provenance information is abbreviated as PI in the figure.

Complete provenance information of any biological material and data (Fig. 5) is important to interpret the data or to enrich an existing biological material and data set consistently. This provenance information must include a link to the source biological material and—if possible—a link to the information on the very research participant who donated the material.

Fragmentation of healthcare information standards, particularly in clinical settings, may pose significant impediments to this process in practice.

For certain cases, it may be necessary to develop robust distributed provenance information schemes, so that on one hand it is possible to reconstruct the whole trail, while also keeping the process compliant with data protection regulations on the other. An example of such a process could be reporting data back in case of incidental findings or if a research participant exercises his/her right to receive the data generated from them. Due to privacy protection requirements, this may involve collaboration between multiple entities responsible for different parts of the provenance information.

Q-1: Provenance information must be FAIR. This involves development and adoption of domain-specific standards for provenance based on commonly accepted provenance information data models.

Q-2: Provenance information must continuously cover the whole chain from sample to data, ideally even from the research participant to sample, while also being compliant with data protection regulations.

### Quality as a prerequisite for extensibility

To develop comparable specimen and data collections, it is important to describe the processes of obtaining and manipulating the sample from the research participant to the storage of the biological material (sometimes informally called “from the needle to the freezer”). For reproducibility reasons, it is also necessary to document all the preanalytical and analytical methods used to generate the data, as has been demonstrated in the literature.^10,17,19^

Q-3: Provenance information must have sufficient technical ability to describe compliance of the biological material with common quality standards, such as preanalytical standards (e.g., ISO or CEN standards).

Q-4: Provenance information must include information about analytical methods and tools used to generate data from the biological material.

## Incentives

In contrast to many other scientific fields, medical data can only be made available to researchers because of voluntary contribution from a variety of individuals, particularly research participants (donors and patients) and medical doctors. A positive incentive scheme must be developed and adopted in wide research communities, which will maximize biological material and data sharing and achieve actual *reuse*. Effective incentive schemes will also exert pressure on resource providers to implement transparent access policies and reduce the fragmentation of access procedures. Similar incentive principles should also be applied to software tools and their sustainability, which is fundamental for any data-driven medical research.

The incentives schemes must implement the following principles:

INCE-1: Incentives must be in place for all the links in the chain: (1) biological material and data generation or collection; (2) biological material and data storage, curation, and enrichment; and (3) biological material and data reuse.

INCE-2: For biological material and data collections receiving public funding or infrastructural funding, the incentive must stimulate reuse by external users, namely users outside the infrastructure.

INCE-3: Contributions to existing biological material and data collections should be supported by funding organizations.

INCE-4: Academic promotion schemes and institutional evaluation schemes should incorporate contributing to and reuse of existing biological material and data collections.

Curation of biological material and data in INCE-1 needs to be understood as a more complex process, and not just the updating of data formats. This includes increasing its extent, as well as the enriching existing biological material and data with other types of material. Similar principles should also be applied to software tools given that medical research is dependent on the long-term availability and maintenance of high-quality software tools for data processing.

Reuse of biological material and data needs to include incentives for both sides, namely providers and accessors. Not only should researchers have incentives to use existing resources but resource providers also need clear incentives to promote and facilitate the reuse of their resources. Enforcing these principles by funding bodies, publishers, and academic organizations will exert pressure on resource providers to implement transparent access policies and make their resources more easily accessible to demonstrate their reuse. Furthermore, this should help to reduce the fragmentation of access procedures, which are effectively preventing reuse and integration of resources on a larger scale, as well as in biobanking.^20,21^

There is ongoing active development of metrics related to these incentive systems, as witnessed by altmetrics** by NISO^††^ or CASRAI,^‡‡^ and proposals to implement transient credit with JSON-LD.^22^ Micro-attribution schemes have also been suggested to support acknowledgment of contributing to large genomics data sets.^23,24^ These are further supported by the development of technical procedures on how to reference resources, such as BRIF^25^ and the CoBRA guideline^26^ for biomedical and health resources. However, to date none of these systems have been widely adopted in practice.

## P: Privacy-Respecting Access

Particularly in the context of human data used in medical research, there are three naturally competing interests: (1) protection of privacy of individuals contributing their personal and potentially privacy-sensitive data; (2) reuse of data to maximize return on investment into research and society; and (3) complex ownership situation and economic interests. These needs have been recognized by various medical communities, as witnessed by the efforts toward clinical trial data sharing.^27^

**FIG. 6.**
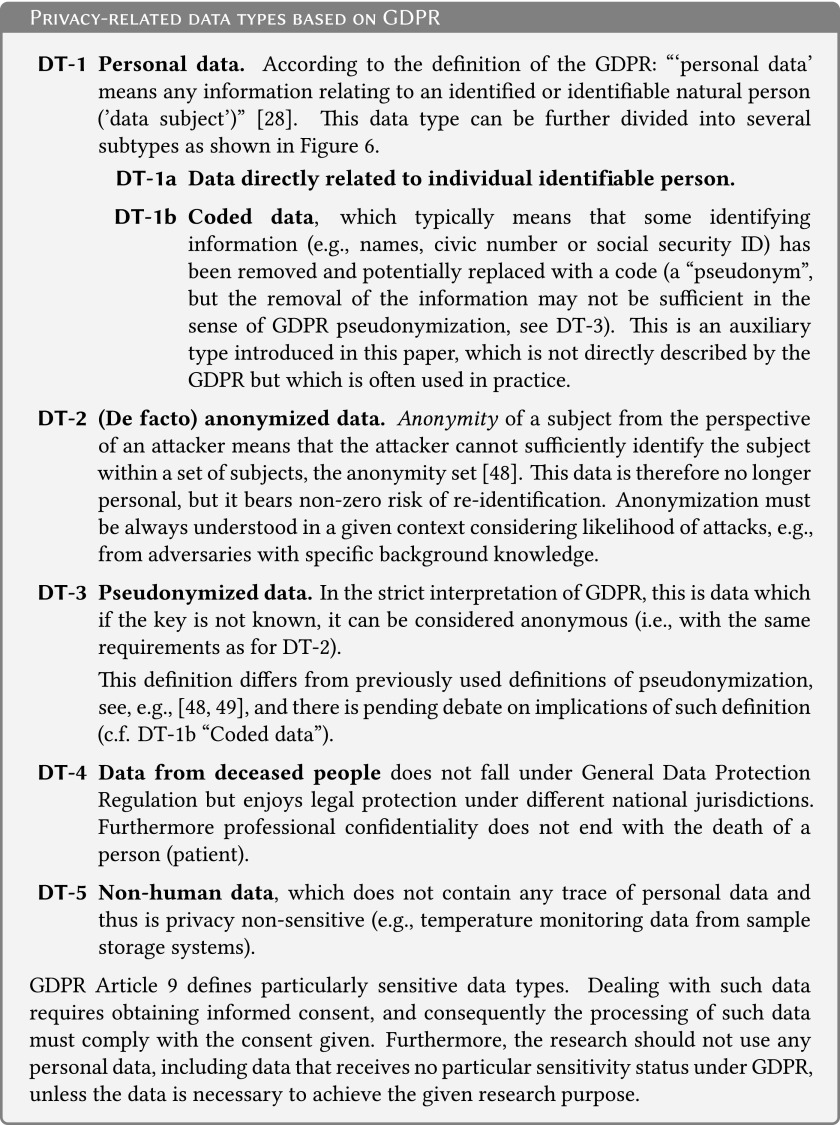
Privacy-related data types based on GDPR. GDPR, General Data Protection Regulation.

As a basis for the discussion of data protection, we need to distinguish basic data types (Figure 6). We use the European General Data Protection Regulation (GDPR)^28^ as a basis, given that it is the most recent regulatory framework with transnational impact and because of international research collaboration, it is very likely to have an impact on a global scale. In the following text, we will also use the term *privacy-enhancing technologies* for a wide range of technologies that protect informational privacy by eliminating or minimizing personal data,^29^ e.g., coding (DT-1b) or anonymization (DT-2).

In the medical research domain, data sets are often anonymized by reducing the precision of attribute values or by removing them entirely,^30^ and a number of practical methodologies and tools doing just this have recently become available.^31,32^ For most people, the notion of anonymity implies that the remaining data are no longer privacy sensitive because no information can be traced back to or derived about the data subjects.^33^ However, this is not the case for many anonymization techniques. Indeed, the frequently used simple *k*-anonymity is, for example, prone to attribute disclosure.^34^ Even differential privacy, which is the only approach that provides rigorous mathematically grounded privacy guarantees, assumes that data cannot be totally anonymous and remain useful at the same time.^35^

Thus, the term (*de facto*) anonymized is preferred and should not be understood as static and binary, but rather as a set of various techniques for *minimizing risks of privacy breaches.*^33^

It needs to be stressed that (*de facto*) anonymization is often not needed, such as in cases where informed consent is available for the given purpose, and other privacy-enhancing technologies, such as coding, may be used in conjunction with additional organizational measures. It is the responsibility of the data custodian to estimate privacy risks and adjust the technical and organizational access conditions and procedures appropriately.

In this context, there are advantages of developing large collections of biological material and data. Often, the greater the number of individuals included in a data set that is to be privacy enhanced, (1) the less information per individual has to be removed or perturbed to achieve the same residual risk of reidentification^30,36^ and (2) the lower the risk of reidentification when the same privacy-enhancing technology is applied.

While authors of the FAIR principles have already assumed that *accessible* does not always imply “open access” we introduce additional requirements to FAIR-Health to avoid overadvertising and support compliance with legal requirements:

PR-1: There must be clear identification of the responsible data controller for any given biological material and data set, who can be contacted by data subjects (research participants) or authorities.

PR-2: Compliance of (intended) research projects with informed consent and the ethics approval of the research project must be evaluated before providing access to sensitive biological material and data.

PR-3: Privacy-enhancing technologies should be applied to personal data (DT-1a) before the data can be used for research purposes, in compliance with the data minimization principle.

PR-4: Before releasing (*de facto*) anonymized data (DT-2), residual privacy risks, including risks of reidentification, must be considered by the data controller. The residual risks must also take into account additional safeguards, such as restricted access with sufficient level of identity assurance.^37,38^

PR-5: Privacy-enhancing technologies should preserve maximum value of data, while keeping the risks at an acceptable level.

The application of privacy-enhancing technologies on data to be released as a whole, as opposed to *per partes* on the source data subsets, should be considered. In many cases, this will minimize effects of transforming data and reduce data perturbations.

PR-6: Data provenance must be implemented in a way that allows for identification of relevant data sets in case of informed consent withdrawal. It should be noted that in case of (*de facto*) anonymous data (DT-2), removal of data from a specific individual may no longer be possible.

PR-7: Specifically in the case of health-related or medical data, informed consent as well as data/material transfer agreements (DTA/MTA) must define policy as to how to address incidental findings and whether access to individual's own data and results is provided, as well as how they will be accessed. In particular, the required technical and organizational safeguards have to be described.

Policies implementing PR-7 should also consider and adequately communicate that application of some privacy-enhancing technologies may prevent communicating incidental findings or providing access to individual's own data/results. This is especially important when a research project uses (*de facto*) anonymized data (DT-2).

Yet, no matter how high the data protection standards are set, there will always remain some risk of (re-)identification of individuals and disclosure of sensitive information about them. What is needed is the tightening of rules to protect against privacy violation that can lead to risks such as discrimination based on genetic information; the U.S. Genetic Information Nondiscrimination Act (GINA) is a good starting point^39^ that can be further elaborated upon.^§§^

PR-8: Legal protection must be developed and implemented for individuals whose privacy has been breached accidentally or unlawfully.

### Privacy-respecting scalable data processing and storage

The recent decade has seen the rise of cloud computing,^40,41^ allowing for various business models. As convenient as the cloud infrastructures are to achieve scalable processing and storage, they bring additional risks when used for processing data.^42,43^ Several modes of operation can be implemented with respect to data protection, where data storage and processing can take place:

1. in *private clouds*^41^ built and operated by the data controller (e.g., a biobank in our case)—this enables processing of any type of data;
2. in an infrastructure that is contracted by way of a third party under such conditions that enable the third-party infrastructure to logically become part of the private infrastructure, thereby operating under the same liability for both data controller and for infrastructure provider—*“logically private clouds”;* and
3. in *public clouds* where the cloud provider does not provide any specific data protection guarantees—this is mostly restricted to data that do not require legal protection, namely (*de facto*) anonymized data (DT-2) with very low risk of reidentification (see requirement PR-4) or nonhuman data (DT-5).

If the data controller agrees to transfer the data to the researcher under a Material/Data Transfer Agreement (MTA/DTA), the researcher has the same modes available. Modes 2 and 3 are nowadays subject to major ongoing development from the data protection perspective. International standards in this field recently emerged, such as ISO 27018.^44^ However, their adequateness and acceptance in medical research are largely open issues, hence the following additional requirements:

PR-9: Commonly-accepted policies and procurement guidelines must be developed under conditions where the third-party infrastructures can logically become private infrastructures suitable for storing and processing privacy-sensitive data.

PR-10: Commonly accepted guidelines must be developed for storing and processing data covered by data protection regulations on public infrastructures.

## Conclusions

Life sciences generally suffer from fragmentation, while medical research in particular suffers also from substantial reproducibility issues. In this article, we proposed to extend the recently developed FAIR principles to FAIR-Health principles related to *quality*—namely reproducibility and meaningfulness—by providing comprehensive provenance information for the complete chain from a donor to biological material to data, as well as *incentives* for enriching existing resources and reusing them. Given the use of human material and data in medical research, we also propose *privacy-protecting principles* related to compliance with data protection regulations. European researchers, in collaboration with experts from multiple domains, including legal experts, computer science experts, and medical researchers, as well as research participants and citizens, are now gathering to define specific rules for a Code of Conduct for GDPR^45^ to ensure compliance with the regulatory frameworks. For medical research, all of these components called FAIR-Health are fundamental prerequisites for effective reuse of biological material and data.

## About BBMRI-ERIC

In 2006, BBMRI was one of six Life science proposals that became part of the European Strategy Forum on Research Infrastructures (ESFRI) Roadmap for Research Infrastructures in Europe. During 2008–2011, BBMRI was funded as an EU project during the preparatory phase. After a transition phase, the Members and Associated States of the European Union approved the infrastructure as a legal entity in 2013, as a European Research Infrastructure Consortium (ERIC). Today BBMRI-ERIC is one of the largest European Research Infrastructure in the field of medicine and health. www.bbmri-eric.eu/

## Notes

^*^ www.bbmri-eric.eu

^†^ www.esf.org/fileadmin/Public_documents/Publications/Code_Conduct_ResearchIntegrity.pdf in particular also deals with data management in Good Research Practice (section 1.4) point 1, and Guidelines for Good Practice Rules (section 2.3) point 1.

^‡^ This behavior has been documented, for example, in EU FP7 SPIDIA project (www.spidia.eu) and has become part of Annex A of CEN/TS 16826-1:2015.^18^

^§^ These developments are already reflected in efforts such as ISO TC 276 (Biotechnology) Working Group 5 on data integration.

^**^ https://en.wikipedia.org/wiki/Altmetrics.

^††^ www.niso.org/topics/tl/altmetrics_initiative.

^‡‡^ www.casrai.org/Dataset_Level_Metrics.

^§§^ GINA provides some protection against discrimination in health insurance, but it does not provide protection against discrimination in life insurance and long-term care insurance.
